# Predation and selection for antibiotic resistance in natural environments

**DOI:** 10.1111/eva.12353

**Published:** 2016-01-25

**Authors:** Jørgen J. Leisner, Niels O. G. Jørgensen, Mathias Middelboe

**Affiliations:** ^1^ Department of Veterinary Disease BiologyFaculty of Health and Medical SciencesUniversity of CopenhagenFrederiksbergDenmark; ^2^ Department of Plant and Environmental SciencesFaculty of ScienceUniversity of CopenhagenFrederiksbergDenmark; ^3^ Department of BiologyMarine Biological SectionFaculty of ScienceUniversity of CopenhagenHelsingørDenmark

**Keywords:** antibiotic resistance, interference‐based competition, nutrient limitation, predation, resource‐based competition

## Abstract

Genes encoding resistance to antibiotics appear, like the antibiotics themselves, to be ancient, originating long before the rise of the era of anthropogenic antibiotics. However, detailed understanding of the specific biological advantages of antibiotic resistance in natural environments is still lacking, thus limiting our efforts to prevent environmental influx of resistance genes. Here, we propose that antibiotic‐resistant cells not only evade predation from antibiotic producers but also take advantage of nutrients released from cells that are killed by the antibiotic‐producing bacteria. Thus, predation is potentially an important mechanism for driving antibiotic resistance during slow or stationary phase of growth when nutrients are deprived. This adds to explain the ancient nature and widespread occurrence of antibiotic resistance in natural environments unaffected by anthropogenic antibiotics. In particular, we suggest that nutrient‐poor environments including indoor environments, for example, clean rooms and intensive care units may serve as a reservoir and source for antibiotic‐producing as well as antibiotic‐resistant bacteria.

## Introduction

**Figure 1:**
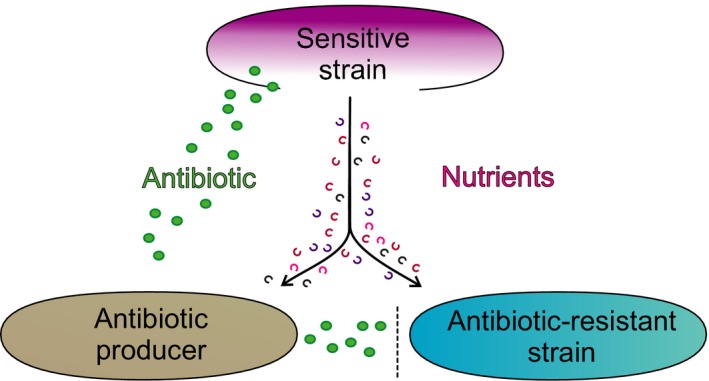
Overview of nutritional effect by antibiotic‐driven predation on antibiotic‐resistant cheater cells.

A substantial amount of literature exists on the phenomenon of selection for bacterial antibiotic resistance, but the mechanisms underlying its emergence and evolution are still not completely understood (Wright 2007; Aminov 2009; Allen et al. 2010; Davies and Davies 2010; Leisner and Haaber 2012; Galan et al. 2013; Khumbar and Watwe 2013). This uncertainty is also mirrored in a similar lack of consensus on the biological role of antibiotics in nature (Aminov 2009; Martinez 2009; Davies and Davies 2010; Hibbing et al. 2010; Galan et al. 2013; Sengupta et al. 2013). General hypotheses for the production of antibiotics in natural environments have focused on competition, signaling, and to a lesser degree on predation. Recently, we suggested predation as a selection mechanism for production of antibiotics, especially in nutrient‐limited environments as antibiotic production by a given strain may secure supply of nutrients from the lysed target cells (Leisner and Haaber 2012; Fig. 1). In a nutrient‐limited scenario, resistance to antibiotics may serve as a defense mechanism against predation, but it may also be a strategy for obtaining nutrients without energetic cost associated with production of antibiotics. In this review, we argue for predation by antibiotics as a selection mechanism that may contribute to explaining the widespread occurrence of antibiotic resistance in natural environments. Such a scenario has implications also for anthropogenic nutrient‐deficient environments such as clean rooms and intensive care units.

## What is the biological function of antibiotics?

There are various explanations to the biological function of antibiotics. Interference‐based competition that involves direct antagonistic interactions between competitors (as opposed to competition on resources; Hibbing et al. 2010) and signaling has received most attention, but predation has also been suggested as an alternative explanation (Table 1). Specifically, antibiotic production and resistance may be a result of predation‐driven selection during a nutritional bottleneck in the late exponential or stationary growth phase. This situation therefore presents a combination of interference‐based and resource‐based competition. It is well known that selection under nutrient‐poor conditions is associated with differences in survival rates that may lead to an improved fitness – probably due to a variety of different parameters – relative to the wild type as observed for long‐term survival of bacterial cultures (Rozen et al. 2009; Eisenstark 2010). Increased survival ability of mutants during nutritional bottlenecks has also been modeled for variants of bacterial pathogens during environmental transmission from one host to another (Handel and Bennet 2008). It should be noted that the latter scenario is not associated with the evolution of virulence, designated as the curse of the pharaoh hypothesis, stating that long‐lived parasites may evolve higher virulence (Bonhoeffer et al. 1996; Gandon 1998). Rather, the improved survival of bacterial cells during starvation at these conditions is related to feeding on lysed competitor cells (Rapposch et al. 1999; Finkel and Kolter 2001; Rozen et al. 2009).

**Table 1:** Examples of suggested biological roles for antibiotics

Antibiotic^a^	Cellular target	Suggested biological role			References
		Predation^b^	Competition	Signaling	
	*Cell wall/membrane*				
Class I, II, III bacteriocins	Cell membrane and/or cell wall	+			Leisner and Haaber (2012)
Gramicidin	Cell membrane	+?^c^			Dubos (1939)
Myxovirescin	Cell wall	+^d^			Xiao et al. (2011)
Pumilacidin	Membrane	+			Brack et al. (2015)
Tyrocidin	Membrane	+^c^			Dubos (1939)
T6SS	Membrane	+			Pukatzki and Provenzano (2013), MacIntyre et al. (2010)
	*Intracellular*				
Actinomycin	Inhibit transcription	+			Waksman and Woodruff (1941)
Colicin E2	DNA		+		Kerr et al. (2002)
Corallopyronin	RNA polymerase	+			Xiao et al. (2011)
Norfloxacin	DNA gyrase			+^e^	Linares et al. (2006)
Tetracycline	30S ribosome			+^e^	Linares et al. (2006)
Tobramycin	30S ribosome			+^e^	Linares et al. (2006)
	*Not defined*				
*Streptomyces* antibiotics		+			Kumbhar et al. (2014)

a Class or distinct type of antibiotic. Bacteriocins and protein nanotubes are designated as antibiotics.

b Only predation that fits with lysis of target cells is shown. Predation among bacteria that does not involve antibiotics as killing of various microorganisms by *Bdellovibrio* (Jonke et al. 2014) is not included.

c Gramicidin did not cause cell lysis in the study by Dubos (1939). It has been suggested that both gramicidin and tyrocidin play a role for sporulation, although there is conflicting evidence regarding this issue (see, e.g., Pschorn et al. 1982; Symons and Hodgson 1982; Piret and Demain 1983).

d It should be noted that lytic factors produced by *Myxococcus xanthus* are released in response to prey cell contact, rather than solely in response to nutritional cues. Targeted and regulated secretion requires lower concentrations of lytic factors (Berleman and Kirby 2009), and therefore, it can be anticipated that the occurrence of antibiotic resistant cheater cells would be minimized in this particular example.

e At antibiotic concentrations < minimum inhibitory concentration.

Here, we propose that one of the roles of antibiotics is to increase survival of both antibiotic producers and resistant cheater cells (i.e., cells that take advantage of nutrients released due to antibiotic‐driven cell lysis) during bottleneck situations, not only for pathogenic bacteria but also for environmental bacteria residing in nutrient‐deprived habitats (Fig. 1). A key assumption in this model is that nutrients released from sensitive target cells are sufficient to sustain growth and survival of the antibiotic producers, as well as of the antibiotic‐resistant cells, in environments with otherwise low levels of nutrients. Dubos' classical study (1939; see further below) points to this as a possibility, but the mechanism has not been further investigated.

An additional function of antibiotics may be signaling among cells and thus acting in quorum sensing communication. However, signal molecules involved in bacterial communication are generally found to occur in miniscule amounts well below the minimum inhibitory concentration and minimum bactericidal concentration of the target cells (Aminov 2009; Davies and Davies 2010; Bernier and Surette 2013), whereas antibiotic‐driven predation is associated with a much higher range of antibiotic concentrations.

## Historical studies suggesting predation as a biological role of antibiotics

Already 75 years ago predation was applied as a methodological principle for isolation of some of the very first antibiotic‐producing organisms, including the gramicidin‐ and tyrocidine‐producing *Bacillus brevis* (Dubos 1939; Dubos and Hotchkiss 1941) and the actinomycin‐producing *Streptomyces antibioticus* (Waksman and Woodruff 1941) (Table 1). In Dubos' study, an intact soil sample was incubated at 30°C for a few weeks to ensure decomposition of the most labile organic matter originally present. Subsequently, living cells of staphylococci and streptococci were added for a couple of years (sic) to the soil sample serving as an organic matter supply for antibiotic‐producing cells originally present in the soil. When the soil microflora was subsequently analyzed, it was found to contain a *Bacillus* population which was capable of lysing a *Staphylococcus* culture due to production of an antibiotic that was later identified as tyrocidine (Dubos and Hotchkiss 1941). One conclusion from this discovery was that the added cultures of staphylococci and streptococci had served as nutrients for the antibiotic‐producing *Bacillus* population, most likely due to antibiotic‐mediated cell lysis. Interestingly, the authors report that the soil sample not only harbored a *Bacillus* population, but also a mixed bacterial flora. Although sensitivity of this community to antibiotics was not reported, it was most likely tolerant or resistant to the antibiotic‐producing *Bacillus* culture, thus belonging to the environmental resistome, that is, the collection of antibiotic resistance genes and their precursors in bacteria in the environment (D'Costa et al. 2006, 2011; Wright 2007; Galan et al. 2013). The mixed bacterial flora observed by Dubos and Hotchkiss may have proliferated by utilizing nutrients released from bacteria that were killed by antibiotics which did not affect the mixed bacterial population. The pioneering work of Dubos and others provided the first indication for a mechanism potentially involved in selection for antibiotic resistance in natural communities of bacteria. In its extreme consequence, this mechanism selects for resistant bacteria that subsist on antibiotics (Dantas et al. 2008).

## Do antibiotics cause predation in pristine natural environments?

Antibiotics must be produced in sufficient quantities to ensure lysis of neighboring sensitive target cells for predation and predation‐driven resistance to function as a survival strategy. Production of antimicrobial compounds in pristine natural environments is expected, because antibiotic‐producing bacteria, for example, members of the *Streptomyces* genus, are widespread in natural environments (Lee et al. 2011). Yet, demonstrating the presence of free antibiotics in water and soil at pristine sites is challenging because many antibiotics react with inorganic and organic compounds, impairing their analytical detection (Tong et al. 2009). Also, degradation by UV light may reduce the concentration of antibiotics in natural environments (Halling‐Sørensen et al. 1998). These mechanisms may explain why tetracyclines (antibiotics produced by various *Streptomyces* species) could not be detected in water of the upper pristine and anthropogenic unaffected section of Cache la Poudre River in Colorado, USA (Yang and Carlson 2003). Further downstream, several microbial‐produced antibiotics (tetracyclines) and synthetic antibiotics (sulfonamides) were detected in the water. The presence of synthetic compounds in the river suggested an anthropogenic discharge of these compounds from farms, sewage treatment plants, and other urban areas, rather than production by indigenous microorganisms. Contrasting these observations on the occurrence of antibiotics in natural environments, Berendsen et al. (2013) recently observed a high production of the antibiotic compound chloramphenicol by a population of added soil bacteria.

These limited observations on production of antibiotics in pristine environments do not allow conclusions on whether antibiotics actually provide nutrients to microbial populations in such environments. It is important to note, however, that a predatory effect of antibiotics does not necessarily require widespread high concentrations of these compounds but may occur in microscale hotspots with an elevated microbial activity. Documentation of the presence of such antibiotic hot spots is technically challenging, due to the lack of methods for high‐resolution detection of antibiotics at microscale levels in natural environments. However, such quantification of antibiotics in the immediate vicinity of antibiotic‐producing cells may clarify if their concentrations exceed the minimum bacteriocidal values necessary to initiate a predation‐mediated lysis.

## Antibiotic resistance offers an advantage during survival in nutrient‐poor environments by allowing utilization of nutrients released from lysed sensitive strains

Maintenance of the ability to resist antibiotics is often expected to be associated with a fitness cost, such as a diminished growth of the bacteria. Documentation for such a fitness expense in antibiotic‐resistant variants has traditionally been provided by analysis of growth performance under nutrient‐rich conditions, that is, during exponential growth in laboratory cultures, and not during nutrient‐limited conditions that often prevail in nature (e.g., Björkholm et al. 2001; Nagaev et al. 2001; Nilsson et al. 2003; Kusuma et al. 2007; Rozen et al. 2007). Differences in growth rates during the exponential phase offer an easy, efficient, and rapid method to explore fitness differences between antibiotic‐sensitive and antibiotic‐resistant variants, for example, when characterizing fitness properties in antibiotic‐resistant variants of pathogenic bacteria during host infection (reviewed by Andersson and Hughes 2010). However, many pathogenic bacteria, as well as nonpathogenic bacteria, also reside in nutritionally sparse environments in nature. In a model for transmission and evolution in pathogenic bacteria, Handel and Bennet (2008) suggested that selection under such circumstances might be driven by fitness parameters associated with survival, rather than selection for increased virulence (Bonhoeffer et al. 1996; Gandon 1998). For pathogenic bacteria, this is relevant in spatially structured environments in which selection favor strains with low degree of transmission and low virulence in static phase (see discussions by Boots and Sasaki 1999; Galvani 2003; Day and Proulx 2004).

There is some experimental evidence that structured habitats that allow formation of antibiotic‐mediated inhibition zones and with increased concentrations of nutrients select for *Escherichia coli* cells that produces an antibacterial bacteriocin (Chao and Levin 1981). Here, we suggest that predation by antibiotic producers and neighboring‐resistant cheater cells can be selected for in nutrient‐poor environments provided that there initially are high amounts of antibiotic‐sensitive cells present, a scenario that differs from those outlined by Chao and Levin (1981). Although several studies demonstrate a possible role of antibiotic production in bacterial predation (Table 1) direct experimental evidence that such predation also favors resistant cells is lacking.

There is work showing that selection on nonsocial traits limits invasion of social cheats (Morgan et al. 2012), a situation that relates to growing or colonizing populations. This is not relevant for populations facing a bottleneck if it is assumed that the antibiotic‐resistant cheater cells initially are present as a minor fraction of the total population and their subsequent relative increase in frequency is due to improved survival and not a result of growth or colonization.

Several antibiotics act only on dividing bacterial cells and therefore have no effect on nondividing vegetative persister cells (Lewis 2010) or bacterial spores. Such nonlysed persisters and/or spores will therefore not support the survival of bacterial vegetative cheater cells that are resistant to but do not produce antibiotics. However, even in conditions with no overall growth (i.e., the stationary phase), the majority of bacterial populations will in most circumstances be composed by a mixture of growing or dying nonpersister cells that are subjected to selection, as demonstrated by the growth advantage in stationary phase mutants observed for *E. coli* and other taxa (Finkel 2006). Under such circumstances and in a nutrient‐poor environment, selection of antibiotic‐resistant cheaters may readily occur as suggested here.

If antibiotic‐mediated predation indeed is a common mechanism for obtaining nutrients in nutrient‐poor environments, selection for antibiotic resistance may be of key importance in such settings. The huge environmental resistome that encompasses bacterial communities found in nutrient‐poor settings, yet unexposed to anthropogenic antibiotics, for example, in caves (Bhullar et al. 2012), may support that such environments selects for antibiotic resistance (Guardabassi et al. 2004; D'Costa et al. 2006, 2011; Cytryn 2013) although, as noted above, there is a lack of studies demonstrating the presence of antibiotics in concentrations sufficient for lysis of target cells. Although resistance to antibiotics *per se* offers a selective advantage in their presence, we argue here that antibiotic‐resistant bacteria may possess an additional increased fitness when nutrients are scarce. Such an improved fitness will result in longer survival than would be the case if the antibiotic‐resistant cells did not benefit from nutrients provided from lysed antibiotic‐sensitive cells. Changes in survival rates are difficult to verify by traditional fitness assays, for example, from changes in growth rate during the exponential phase. Implementation of alternative analytic techniques such as detection of fluorescent marker genes by flow cytometry may allow for detection of very small fitness effects (Gallet et al. 2012). Even so, it is not clear to what extent increased availability of additional resources (from predation) represent a significant fitness gain relative to the benefit of eliminating the risk of being killed by the antibiotic. It can be anticipated that the fitness value associated with utilizing nutrients of lysed cell materials might add up with time. For this reason, survival experiments for determination of fitness require a relatively long incubation time.

We argue therefore for the need for more detailed characterization of fitness characteristics associated with antibiotic resistance in nutritional‐sparse environments. It is important that such studies are designed in order to eliminate fitness effects that are not necessary related to the issue of nutrients such as the introduction of compensatory mutations (Levin et al. 2000) or horizontal gene transfer events.

## Lysis of target cells is a prerequisite for predation

Lytic effects of antibiotics produced can be challenging to observe in experimental settings, because lysis is not always an immediate reaction following exposure to the antibiotic. For example, some bacteriostatic antibiotics are only bacteriocidal after a longer period of exposure (typically measured in days), as observed for bacteriocins produced by lactic acid bacteria (Leisner and Haaber 2012). A delayed lysis of the target cells means that the antibiotics may not have an instant effect, but may secure supply of nutrients at a later stage, for example, when the population bottleneck is more severe. A delayed lysis may be due to the antibiotic‐mediated activation of prophages or, more frequently, caused by peptidoglycan‐degrading autolysins. For example, autolysins produced by lactic acid bacteria appear to explain the delayed antagonistic effect of bacteriocins typically observed by these bacteria (summarized by Leisner and Haaber 2012).

## Lack of studies on predation in natural habitats: case study on *Pseudomonas* spp.

Studies on antimicrobial interactions in natural, complex microbial communities have primarily focused on effects on growth rates of antibiotic‐producing as well as sensitive and resistant target cells, rather than on survival rates during nutrient‐limited conditions (e.g., Vetsigian et al. 2011; Cordero et al. 2012). Positive effects on growth and/or survival of antibiotic‐producing and antibiotic‐resistant bacteria in natural environments are difficult to document. For example, addition of an antibiotic‐producing *Pseudomonas* strain to a natural organic‐rich rhizosphere soil had a minor effect on the indigenous bacterial populations (Naseby et al. 2001; Viebahn et al. 2003), suggesting that the antibiotics did not cause lysis‐induced release of nutrients in favor of the *Pseudomonas* strain. Possibly, antibiotics appear in such surroundings more likely to protect the producers from invasion and/or to act on neighbors by signaling as the antibiotic concentrations were low (Haas and Keel 2003; Raaijmakers and Mazzola 2012). A high content of nutrients as typically found in the rhizosphere (Berendsen et al.^2012^; Philippot et al. 2013) may also have reduced the need for predation by the *Pseudomonas* strain. Further, antagonistic activity from antibiotics may have a patchy occurrence in soil microsites (Raaijmakers and Mazzola 2012). Finally, it should be noted that the majority of antibiotics produced by *Pseudomonas* spp. isolates from the rhizosphere appear to mainly target fungi (Haas and Keel 2003). The study on *in situ* production of antibiotics by *Pseudomonas* spp. emphasizes the complexity in examining microscale antimicrobial effects and suggests that studies on the predatory role of antibiotics and predation‐driven antibiotic resistance should target nutrient‐poor rather than rhizosphere environments and be performed at a high spatial resolution, covering the scale of interactions between individual cells and/or small populations of cells.

## Synergy effects between phages and antibiotics

Antimicrobial effects from antibiotics may be enhanced by phage‐mediated stimulation of antibiotics, known as phage‐antibiotic synergy (PAS). PAS has been shown to increase the cell‐lysing effects of antibiotics, even at low antibiotic concentrations. PAS is not restricted to antibiotics targeting cell membrane and/or cell wall synthesis, such as cefotaxime and *β*‐lactams (Comeau et al. 2007; Ryan et al. 2012), but can be triggered also by antibiotics inhibiting cell division, for example, by binding to DNA (mitomycin C; Comeau et al. 2007), or by inhibiting protein synthesis (tetracycline; Santos et al. 2009). Thus, the PAS effect widens the repertoire of antibiotics that can be used in a predatory context. The classical example of PAS is mitomycin C that induces prophage lysis in bacteria (e.g., Weinbauer and Suttle 1999), but other studies have also shown bacteriophages to increase the degree of lysis mediated by antibiotics (Comeau et al. 2007; Santos et al. 2009; Ryan et al. 2012). It would be of interest to investigate whether PAS might add to selection for antibiotic resistance and also what possible role resistance to phage infection might play in that context.

## Practical implications

Among the diverse biological functions of antibiotics, predation appears as an important candidate for selection for antibiotic resistance in nutrient‐poor environments. Predation and selection for cheater strains (i.e., resistant variants that take advantage of lysed nutrients but do not produce antibiotics themselves) may contribute to explaining why antibiotic resistance is an ancient phenomenon, originating long before the era of anthropogenic antibiotics. The medical and veterinary applications of antibiotics have, however, introduced a selective pressure for resistance in nutrient‐rich environments during active infections, characterized by a high growth rate. Thus, new resistance genes may appear which are selected for, or equally important, resistance genes, which have already been selected for in the environment (e.g., soil) will be favored by the new selection pressure.

We argue that a renewed attention on predation and predation‐driven selection for antibiotic resistance can improve our understanding of the mechanisms behind the prevalent occurrence of antibiotic resistance in the natural environment as well as its transfer to bacterial species of medical importance (Forsberg et al. 2012). Predation‐mediated selection for antibiotic resistance may play a role in nutrient‐poor anthropogenic settings, for example, in hospital interiors and equipment, as well as in well‐cleaned domestic surroundings, thus increasing the risk for horizontal transfer of resistance genes to pathogenic bacteria (Wright 2010). Specifically, studies on whether resistant bacteria may have an increased fitness during nutrient‐limited conditions in nutrient‐deficient indoor environments, for example, hospital interiors and equipment, are now called for. Such studies should be designed to measure fitness differences in terms of survival rates rather than exponential growth rates.

## Conflict of interest

The authors declare no conflict of interest.

## Data archiving statement

This study does not contain original data for archiving.
